# Establishment of 3D Co-Culture Models from Different Stages of Human Tongue Tumorigenesis: Utility in Understanding Neoplastic Progression

**DOI:** 10.1371/journal.pone.0160615

**Published:** 2016-08-08

**Authors:** Sharada Sawant, Harsh Dongre, Archana Kumari Singh, Shriya Joshi, Daniela Elena Costea, Snehal Mahadik, Chetan Ahire, Vidhi Makani, Prerana Dange, Shilpi Sharma, Devendra Chaukar, Milind Vaidya

**Affiliations:** 1 Vaidya Laboratory, Advanced Centre for Treatment, Research and Education in Cancer (ACTREC), Tata Memorial Centre, Kharghar, Navi Mumbai, Maharashtra, India; 2 Gade Laboratory for Pathology, Department of Clinical Medicine, and Center for International Health, Department of Global Public Health and Primary Care, University of Bergen, Bergen, Norway; 3 Department of Pathology, Haukeland University Hospital, Bergen, Norway; 4 Nups and Sumo Biology Group, Department of Biological Sciences, Indian Institute of Science, Education and Research, Bhopal, Madhya Pradesh, India; 5 Oral Surgery, Head and Neck Unit, Tata Memorial Hospital (TMH), Parel, Mumbai, India; University of Sheffield, UNITED KINGDOM

## Abstract

To study multistep tumorigenesis process, there is a need of *in-vitro* 3D model simulating *in-vivo* tissue. Present study aimed to reconstitute *in-vitro* tissue models comprising various stages of neoplastic progression of tongue tumorigenesis and to evaluate the utility of these models to investigate the role of stromal fibroblasts in maintenance of desmosomal anchoring junctions using transmission electron microscopy. We reconstituted *in-vitro* models representing normal, dysplastic, and malignant tissues by seeding primary keratinocytes on either fibroblast embedded in collagen matrix or plain collagen matrix in growth factor-free medium. The findings of histomorphometry, immunohistochemistry, and electron microscopy analyses of the three types of 3D cultures showed that the stratified growth, cell proliferation, and differentiation were comparable between co-cultures and their respective native tissues; however, they largely differed in cultures grown without fibroblasts. The immunostaining intensity of proteins, viz., desmoplakin, desmoglein, and plakoglobin, was reduced as the disease stage increased in all co-cultures as observed in respective native tissues. Desmosome-like structures were identified using immunogold labeling in these cultures. Moreover, electron microscopic observations revealed that the desmosome number and their length were significantly reduced and intercellular spaces were increased in cultures grown without fibroblasts when compared with their co-culture counterparts. Our results showed that the major steps of tongue tumorigenesis can be reproduced *in-vitro*. Stromal fibroblasts play a role in regulation of epithelial thickness, cell proliferation, differentiation, and maintenance of desmosomalanchoring junctions in *in-vitro* grown tissues. The reconstituted co-culture models could help to answer various biological questions especially related to tongue tumorigenesis.

## Introduction

All normal tissues rely on a continuous sequence of cellular interactions in a microenvironment comprising several growth factors, hormones, adhesion molecules and an intricate extracellular molecular matrix for their growth and maintenance [1]. Abnormalities in epithelial–mesenchymal interactions lead to a variety of pathological conditions such as premalignant lesions, dysplasia, or even malignancy [2]. Identification of stromal components associated with epithelial tumour invasion process could be useful for controlling pathological growth and the process of tumour invasion and metastasis [3]. Stromal microenvironments include extracellular matrix (ECM), stromal cells such as fibroblasts, adipose cells, resident immune cells, vasculature, cytokines, growth factors etc and they have been shown to have regulatory roles in epithelial cell growth and differentiation [4].

Conventional 2D monolayers and organ cultures have been for long the most popular *in-vitro* models for cancer research. Better viability of cells can be achieved using 2D monolayer culture models and also they are simple and suitable to set up, however, they lack the 3D microenvironment of native tissue. It is known that cellular interactions are essential for gene expression and behaviour of the involved cells [5]. Therefore, the concept of the multicellular 3D models was developed to overcome these shortcomings. 3D cultures support co-cultivation and crosstalk of multiple cell types, such as interacting epithelial and stromal cells, which regulate normal and neoplastic development. They can mimic the tumour–stromal cell interactions of human carcinomas and allow for systematic investigation into the several unidentified regulatory feedback mechanisms in a well-defined 3D milieu [6, 7].

Previous researchers have shown that *in-vitro* 3D models of the various epithelia of the oral cavity can provide a better understanding of oral tissue biology [8, 9], oral mucosal lesions [10–12], gingivitis [13], and other diseases of oral mucosa [14, 15]. In the past, co-culture models were established by various research groups by growing keratinocytes seeded onto the fibroblast-populated matrix and cultured at the air–liquid interface. The reconstructed tissue, thus formed, nearly resembled the native counterpart [16–18]. Previous studies also revealed that fibroblasts have a more profound influence on epithelial morphogenesis as well as neoplastic transformation and progression [8, 19, 20]. To study the contribution of fibroblasts in such epithelial processes, investigators have reconstituted *in-vitro* epithelial tissues using normal and malignancy associated fibroblasts and their findings suggest that malignancy associated fibroblasts actively contribute to malignant progression of neoplastic epithelium [21–24]. Subsequently, researchers developed models in different formats, depending on the scientific question or downstream use of the model, such as to study the early stages of metastasis, angiogenesis, the tumor microenvironment, and cancer stem cells [25]. So far, it has been shown that co-culture models aid in understanding the cell–cell and cell–ECM interactions, along with the effects of the microenvironment on cellular differentiation, proliferation, apoptosis, and gene expression [1].

Oral tumorigenesis is a multistep phenomenon comprising tumour cell initiation, promotion, and progression. Tongue and buccal mucosal cancer are most predominant among the various sub-sites of human oral cavity in Indian subcontinent owing to typical habits of chewing tobacco [26]. Invasion of tumour in the adjacent tissue is very common in both tongue and buccal mucosal malignancies. In order to study the invasive characteristics of the cell, and to understand the process of neoplastic progression during the various steps of oral tumorigenesis, there is a need of some *in-vitro* 3D model system that may represent the *in-vivo* tissue. Based on this, in the present study, we have established 3D organotypic co-cultures (OC) representing *in-vivo* tongue tissues of normal, dysplasia, and malignant using keratinocytes and fibroblasts from the same patient, thus forming true pair.

In the process of oral tumorogenesis, one of the major steps is cell invasion which is aided by reduced cell adhesion. Cell adhesion is mediated by anchoring junctions, gap junctions and tight junctions. Desmosomes, type of anchoring junctions, are composed of proteins from three gene families: Transmembrane cadherins (desmoglein and desmocollin), armadillo (plakoglobin and plakophilin), and plakin (desmoplakin). In addition to mediating cell adhesion, desmosomes are responsible for linking the intermediate filaments of one cell to its adjacent cell [27]. Under TEM, desmosomes can be seen as two-electron dense plaques lying parallel to the adjacent plasma membranes. The desmoplakin protein spans these plaques. The cadherins join to the desmoplakin through plakoglobin and plakophilin with the help of their cytoplasmic domains, which forms the outer dense plaque. The attachment of desmoplakin and the intermediate filaments of the cell is observed in the inner dense plaque [28]. Present study is a first attempt to reconstitute *in-vitro* tissues from isolated primary keratinocytes and fibroblasts of freshly collected tissues from various neoplastic progressive stages of human tongue. Furthermore, we have evaluated the role of stromal fibroblasts in maintaining molecular and structural integrity of desmosomal anchoring junctions using OC grown with fibroblasts (Fib+) and without fibroblasts (Fib-).

We report successful reconstitution of OC models representing various steps of neoplastic progression of tongue tumorigenesis. Our results indicate that stromal fibroblasts exert an influence in the maintenance of desmosomal anchoring junctions in epithelial tissues. We are optimistic that, in future, *in-vitro*-reconstituted tongue tissue models will be useful tool in addressing a broad range of unanswered questions in oral pathogenesis and, especially, in multistep tongue tumorigenesis.

## Materials and Methods

### Collection of Tissue Samples

Human tongue squamous cell carcinoma tissues (*n* = 70) along with their respective surrounding uninvolved normal tissues (1cm farther from the malignancy-free cut margins) and punch biopsies of leukoplakic tongue lesions with a histologically confirmed diagnosis of dysplasia (*n* = 25) were collected from patients undergoing surgical resection and from outpatient department, at Advanced Centre for Treatment, Research and Education in Cancer (ACTREC), Kharghar, India, and Tata Memorial Hospital, Parel, India. Tissues were collected in Dulbecco’s modified Eagle’s medium (DMEM; Gibco, Invitrogen, Carlsbad, CA-US) containing 10% fetal bovine serum (FBS; Hyclone; Gibco, NY, US) and cocktail of antibiotics [100 U/mL penicillin, 100 μg/mL streptomycin, and 1% amphotericin B (Gibco, NY-US)]. Each tissue sample was cut into two parts; one part was used for primary keratinocytes and fibroblasts isolation, while the other part was fixed in 10% neutral buffered formalin and processed for paraffin embedding. Clinicopathological information of patients with OSCC and grade of the dysplasia is given in S1 Table. This study was approved by the Human Ethics Committee of the Institutional Review Board. Written informed consent was obtained from all the subjects before enrolling them in this study.

### Isolation of Primary Tongue Keratinocytes and Fibroblasts

Tissues were washed 4–5 times with phosphate buffer saline (PBS) containing 10% cocktail of antibiotics (100μL cocktail of abovementioned antibiotics and 900μL PBS). Primary keratinocytes and fibroblasts were isolated by direct explant culture method. The tissue was cut into pin-head size pieces with surgical blade and placed in the culture plates flooded with DMEM + 10% FBS. On the basis of the microscopic observations of morphological appearance of the emerging cells, the explants with homogenous outgrowth (either fibroblast-like or epithelial-like morphology) were marked, detached from the original dish, and sub-cultured separately in a fresh culture plate containing keratinocyte–serum-free medium (KSFM, GIBCO Life Technologies, NY-USA) for the growth of keratinocytes and DMEM +10% FBS for the growth of fibroblasts. Both fibroblast-like and keratinocyte-like cells were sub-cultured four to five times before they were used for 3D models. Any cross contamination was eliminated by partial trypsinization-30 second treatment with 0.05% trypsin and 0.02% EDTA followed by vigorous pipetting of the medium. This procedure left epithelial cells adherent to the surface of the culture plate. Further, these cells were trypsinized for 7 min by treating with 0.05% trypsin and 0.02% EDTA at 37°C. In addition, it was observed that fibroblasts were unable to grow in KSFM. Later the outgrown cells were characterized for the expression of keratin and vimentin using immunofluorescence staining technique.

### Preparation of the Simple Collagen Matrix without Fibroblast

The collagen matrix was made as previously described [8] by mixing on ice: 444 μL of rat tail collagen type 1 (3.40 mg/mL, BD Biosciences, MA-USA), 64 μL of 10X DMEM, 128 μL of reconstitution buffer (RB) pH 8.15 [2.2 g NaHCO_3_, 0.6 g NaOH, and 4.766 g 4-(2-hydroxyethyl)-1-piperazineethanesulfonic acid in 100 mL dH_2_O], and 64 μL of FBS per well of 24-well culture plate (BD Falcon, NJ-USA). The pH was adjusted to 7.2–7.4 by using RB. Seven hundred μL of this collagen matrix mixture was layered uniformly in each well of 24-well culture plate. The matrix was allowed to polymerize for 1h in a humidified chamber at 37°C and then equilibrated with 1 mL of routine fibroblast culture medium.

### Preparation of the Collagen Matrix with Fibroblasts

The primary fibroblasts were trypsinized and resuspended in FBS. The collagen matrix was made by mixing on ice: 444 μL of rat tail collagen type 1, 64 μL of 10XDMEM, 128 μL of RB buffer and fibroblasts resuspended in 64 μL of FBS in order to achieve final concentration of 0.25 × 10^6^ fibroblasts/mL of collagen matrix. Fibroblasts were added only after pH neutralization (pH of 7.2–7.4) by using RB buffer. Seven hundred μL of this collagen matrix mixture was layered in each well of 24-well culture plate. The matrix was allowed to polymerize for 1h in a humidified chamber at 37°C and then equilibrated with 1 mL of routine fibroblast culture medium.

### Seeding of Keratinocytes, Lifting, and Maintenance of the 3D Organotypic Co-Cultures (OC)

Before seeding primary keratinocytes, the equilibration medium was removed completely. About 0.5 × 10^6^ keratinocytes in 1mL of KSFM were layered on top of each collagen matrix. After 24h, half of the culture medium was replenished with serum-free organotypic (FAD) medium (3:1 volume of DMEM + Ham’s F12 medium, (Sigma St. Loius, MO-USA)), supplemented with 0.4 μg/mL hydrocortisone (Sigma St. Loius, MO-USA), 5 μg/mL insulin (GIBCO, NY-USA,), 20 μg/mL transferrin (GIBCO, NY-USA), 50 μg/mL L-ascorbic acid (Sigma St. Loius, MO-USA), 200 U/mL penicillin, 200 μg/mL streptomycin, and 0.5 μg/mL amphotericin B. Next day, the matrix was released from the walls of well and was transferred on lens paper (soaked in FAD medium), which was placed on metal grids in a 6-well culture plate (BD Falcon, NJ-USA). Cultures were allowed to grow for next 5–6 days submerged in FAD medium. The cultures were lifted to the air–liquid interface and grown for 7–9 more days. Half of the culture medium was changed every second day during the time of culture growth to maintain endogenous conditioning.

### Harvesting and Processing of OCs

All OCs were harvested on days 12–16. Each OC tissue was divided into 2 parts. One part was fixed in 3% glutaraldehyde and processed for TEM. Second part was fixed in 4% formaldehyde solution and processed for paraffin embedding. Five-micrometer thick sections were cut and stained with hematoxylin and eosin to observe their morphology and to perform morphometry analysis. Immunohistochemistry (IHC) was performed on remaining sections to localize and semi-quantitate protein expression.

### Histopathology and Morphometry

Measurement of the thickness of stratified epithelium was performed on hematoxylin-and-eosin–stained sections using ImageJ software. The “set scale” command was used to calibrate measured distance in pixels against the known distance as seen in the scale bar embedded in the image. All images were calibrated, and the obtained distance in pixels was converted into micrometers. For the measurement of thickness of stratified epithelium of normal and dysplastic OCs along with their respective native tissues, a straight line was drawn from the basement membrane to the uppermost cell layer. The mean of three measurements of different areas of each section was taken as thickness of stratified epithelium. Statistical analysis of the data was done using two-tailed unpaired *t-test* with confidence interval of 95%. The results were expressed as mean ± standard error of the mean (SEM).

### Dual Label Immunofluorescence

Immunofluorescence staining was performed on isolated keratinocytes and fibroblasts as described previously [29]. Briefly, after methanol fixation and permeabilization, cells were incubated with bovine serum albumin (Sigma St. Loius, MO-USA) to block non-specific binding. Further, cells were incubated with primary pan anti-cytokeratin antibody and anti-vimentin antibody for 1h at room temperature (RT; Table 1). After PBS washes, cultures were incubated with Alexa Fluor 488 anti-mouse and Alexa Fluor 568 anti-rabbit secondary antibodies (Thermo Fisher Scientific, IL, USA) for 1h at RT. After counterstaining with 4', 6-diamidino-2-phenylindole (DAPI), nucleic acid dye, sections were mounted and viewed under Zeiss LSM 510 (Zeiss, Germany).

**Table 1:** List of antibodies used in immunohistochemistry /immunofluorescence/immunogold staining protocols.

Antibody	Clone	IgG subclass	UsedConcentration	Working dilution	Supplier/reference
Pan cytokeratin	PCK-26	IgG_1_	50ng/mL	1:200	Sigma Aldrich, MO, USA
Vimentin	HPA001762	IgG	5ng/mL	1:200	Sigma Aldrich, MO, USA
PCNA	PC10	IgG_2a_	10ng/mL	1:50	Nova castra, Germany
Involucrin	SY5	IgG_1_	10μg/mL	1:50	Ad Serotec, NC, USA
Desmoplakin I/II	H-300	IgG	2.8μg/mL	1:70	Santa cruz biotechnology, Germany
Desmoglein	EPR6768	IgG	2.9μg/mL	1:70	abcam, MA, USA
Plakoglobin	4C12	IgG_1_	50ng/mL	1:200	abcam, MA, USA
Alexa Fluor 488-Anti Mouse	-	IgG (H+L)	10μg/mL	1:200	ThermoFisher Scientific,IL, USA
Alexa Fluor 568-Anti-Rabbit	-	IgG (H+L)	10μg/mL	1:200	ThermoFisher Scientific,IL, USA
Anti-Rabbit–Gold secondary antibody	-	IgG (whole molecule)	100μg/mL	1:10	Sigma Aldrich, MO, USA

### Immunohistochemistry

IHC was performed on native tissues and on OCsas mentioned elsewhere [30]. Paraffin sections were first deparaffinized, rehydrated, and then micro-waved in sodium-citrate buffer (pH 6.0) for antigen retrieval. Sections were blocked for endogenous peroxidase activity followed by incubation with pre-immune serum. Sections were further incubated with primary antibodies (Table 1). Next, the sections were incubated with secondary antibody coupled with biotin, followed with the tertiary reagent: avidin–biotin–peroxidase complex (Vector Laboratories, CA, USA). The enzyme–substrate reaction was developed with diaminobenzidine (DAB) chromophore (D5367; Sigma, St. Louis, USA). Counterstaining was done with Harris hematoxylin. Serum from non-immunized mouse/rabbit was used as negative control.

IHC digital images were acquired using the Zeiss ImagerZ1 upright microscope (Zeiss, Germany) equipped with an AxioCam MRc5 camera (Zeiss, Germany). IHC staining intensity score was calculated using ImageJ processing software as described previously [31].

### Quantification of Invasive Depth in malignant Fib+ OCs

Analysis of invasive depth of malignant Fib+ OCs was performed on pan anti-cytokeratin (Table 1) immunostained images which were digitally recorded with a Zeiss Axio Imager Z2 microscope connected to coolcube1 camera using metaviewer V2.0 software. The depth of invasive cells was determined as mentioned elsewhere [32]. Briefly, a day 6 formalin-fixed immunostained Fib+ OC was taken as reference for determining non-invasive cell layer. Further, the distance of the deepest invading cell from the lower surface of this non-invasive cell layer was measured in Fib+ OC grown for 16 days using ImageJ software. Difference in the depth was considered as invasive depth. The mean of three measurements of different areas of each section was taken as invasive depth.

### Transmission Electron Microscopy

For ultrastructural studies, native tissues and harvested OCs were fixed in 3% glutaraldehyde in 0.1M sodium cacodylate–HCL (pH, 7.4) for 2h at 4°C, followed by 1% osmium tetroxide for 1h at 4°C (Ted Pella, Inc., USA). After dehydration, the tissue bits were embedded in Araldite 502 resin (Ted Pella, Inc, USA.) and polymerized. Ultrathin sections (70nm) were cut on ultramicrotome (Leica UC7, Germany) and were obtained on formvar-coated copper grids. Finally, sections contrasted using uranyl acetate and lead citrate and were viewed under Jeol 1400 plus TEM (Japan) at 120 KV.

Analyses of number and length of desmosomes along with intercellular spaces in all the three OC models and their respective native tissues was done using integrated iTEM software (Olympus Soft Imaging Solutions, GmbH, Germany).

### Immunogold Labeling

Ultrathin sections from 3% glutaraldehyde and 1% osmium tetroxide-fixed tissues were collected on nickel grids. Subsequently, the grids were micro-waved in heat-induced antigen retrieval (HIAR) buffer (20mMTris-HCl, pH 9.0) for antigen retrieval as mentioned elsewhere [33]. After blocking with 5% BSA, the sections were incubated with anti-desmoplakin antibody (Table 1) for 1h at RT. Following washes with 1× PBS, grids were incubated with secondary antibody conjugated to 10nm gold nanoparticles (Table 1) for 1h at RT. After thorough washes, the grids were contrasted with uranyl acetate and lead citrate as mentioned earlier and viewed under Jeol 1400 plus TEM (Japan) at 120 KV.

## Results

### Isolation and Characterization of Primary Keratinocytes and Fibroblasts from Native Tongue Tissues

**Fig 1:**
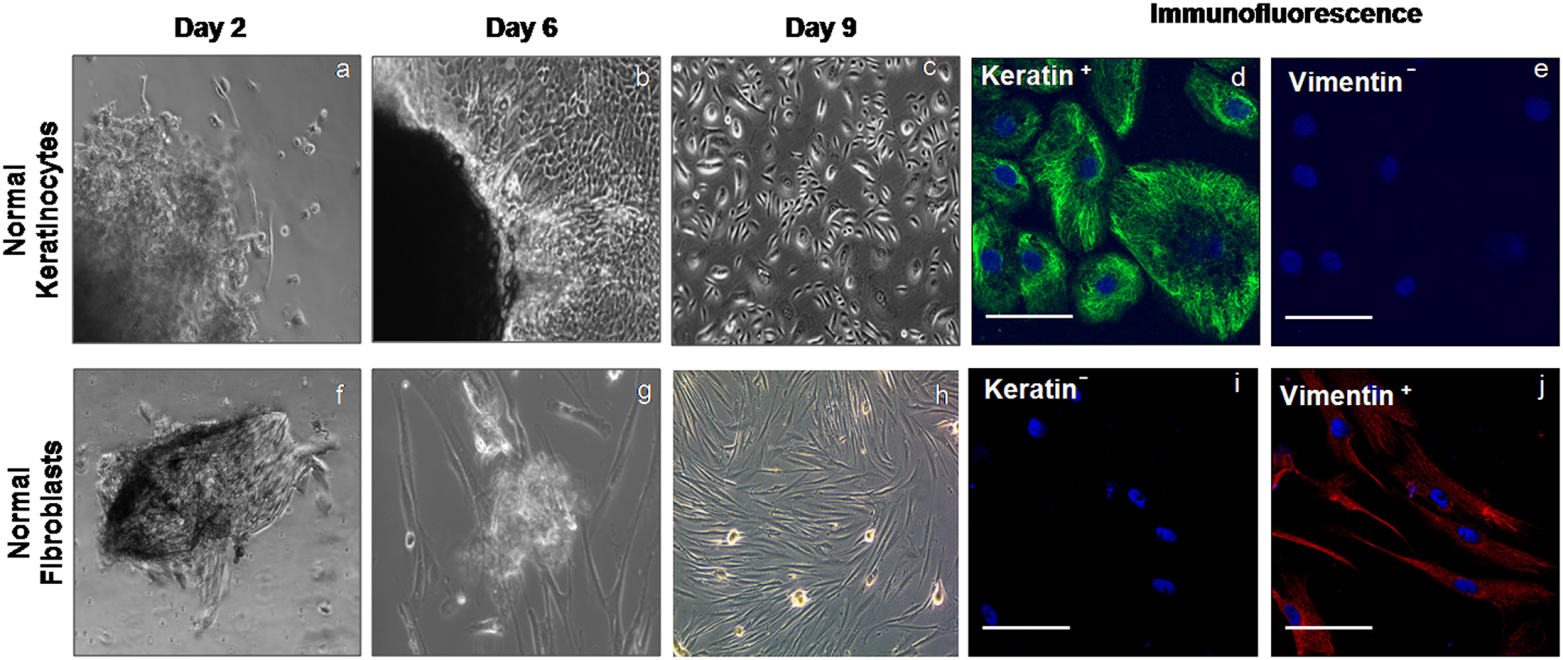
Isolation and characterization of primary keratinocytes and fibroblasts from tongue tissue. Representative phase contrast microscopic images showing the growth of primary keratinocytes/fibroblasts from human normal tongue tissues at different time points–day 2 (a, f), day 6 (b, g) and day 9 (c, h). Immunofluorescence stained images showed the positive expression (green) for cytokeratin (d) and negative expression for vimentin (e) in normal keratinocytes while, fibroblasts showed negative expression for keratin (j) and positive expression (red) for vimentin (i). Nuclei are counterstained with DAPI (blue). Bars-50 μm.

Primary keratinocytes and fibroblasts started emerging out from the explant cultures on day-2. The phase-contrast microscopic images at different time points of growth of cultured keratinocytes and fibroblasts from normal tongue tissues are shown in Fig 1a–1c and 1f–1h, respectively. It was observed that, at about the same time point, keratinocytes and fibroblasts started emerging out from dysplastic and malignant tongue tissues. At day 9, primary keratinocytes and fibroblasts were characterized for the expression of keratin and vimentin using immunofluorescence staining. Normal primary keratinocytes showed positive staining for cytokeratin antibody, while they were negative for vimentin (Fig 1d and 1e). Primary fibroblasts showed negative staining for cytokeratin and positive for vimentin (Fig 1i and 1j).

We obtained pure primary keratinocytes and fibroblasts from 33% (n = 23/70) and 27% (n = 19/70) of malignant and their normal tissues respectively, while in case of dysplastic tissue we got pure primary keratinocytes and fibroblasts from 69% (n = 9/13) tissues. Further, successful growth of 3D models was obtained from 57% (n = 13/23) of malignant, 68% (n = 13/19) of normal and 56% (n = 5/9) of dysplasia tissues. Generally we found better growth of 3D cultures in case of patients with age <40 years.

### Effect of Fibroblasts on Epithelial Thickness

**Fig 2:**
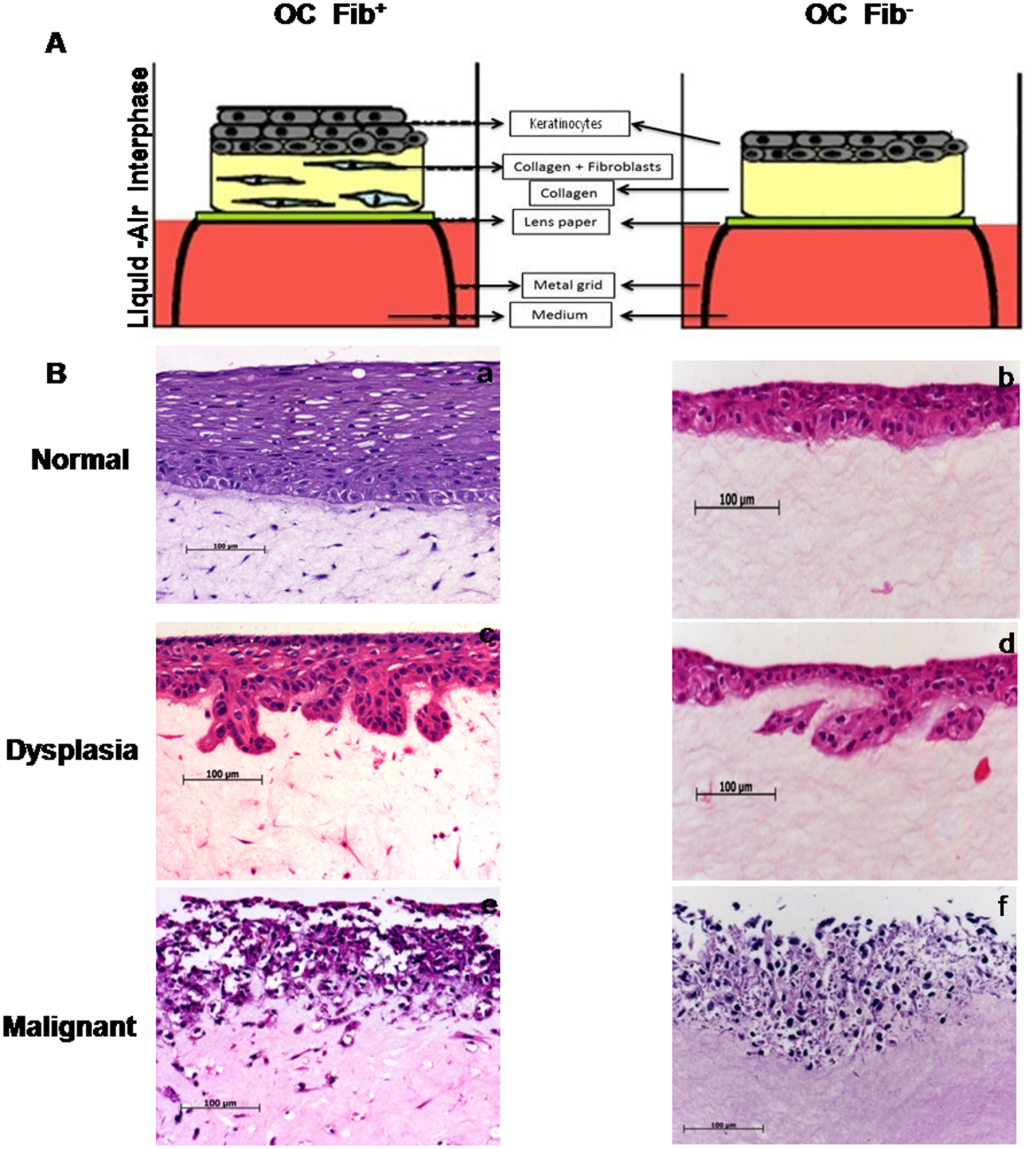
**(A) Schematic diagram of growth of cultures at air-liquid interface:** Keratinocytes are seeded on top of fibroblasts embedded in collagen matrix or plain collagen matrix. Matrix is placed on top of porous paper which is laid on metal grid. The porous paper infuses medium to the culture allowing the differentiation of the tissue to form multilayered epithelium. Presence of fibroblasts enhances the thickness of epithelium. **(B) Images of Hematoxylin and Eosin stained OC sections.** Representative images showing the histomorphology of cultures grown with fibroblasts and without fibroblasts depicting normal (a, b), dysplasia (c, d) and malignant (e, f) respectively. Presence of fibroblasts increased the invasiveness of keratinocytes (e), whereas in the absence of fibroblasts, the cells remained in the epithelial compartment (f). The images are representative of normal (n = 13), dysplasia (n = 5) and malignant (n = 13).

**Fig 3:**
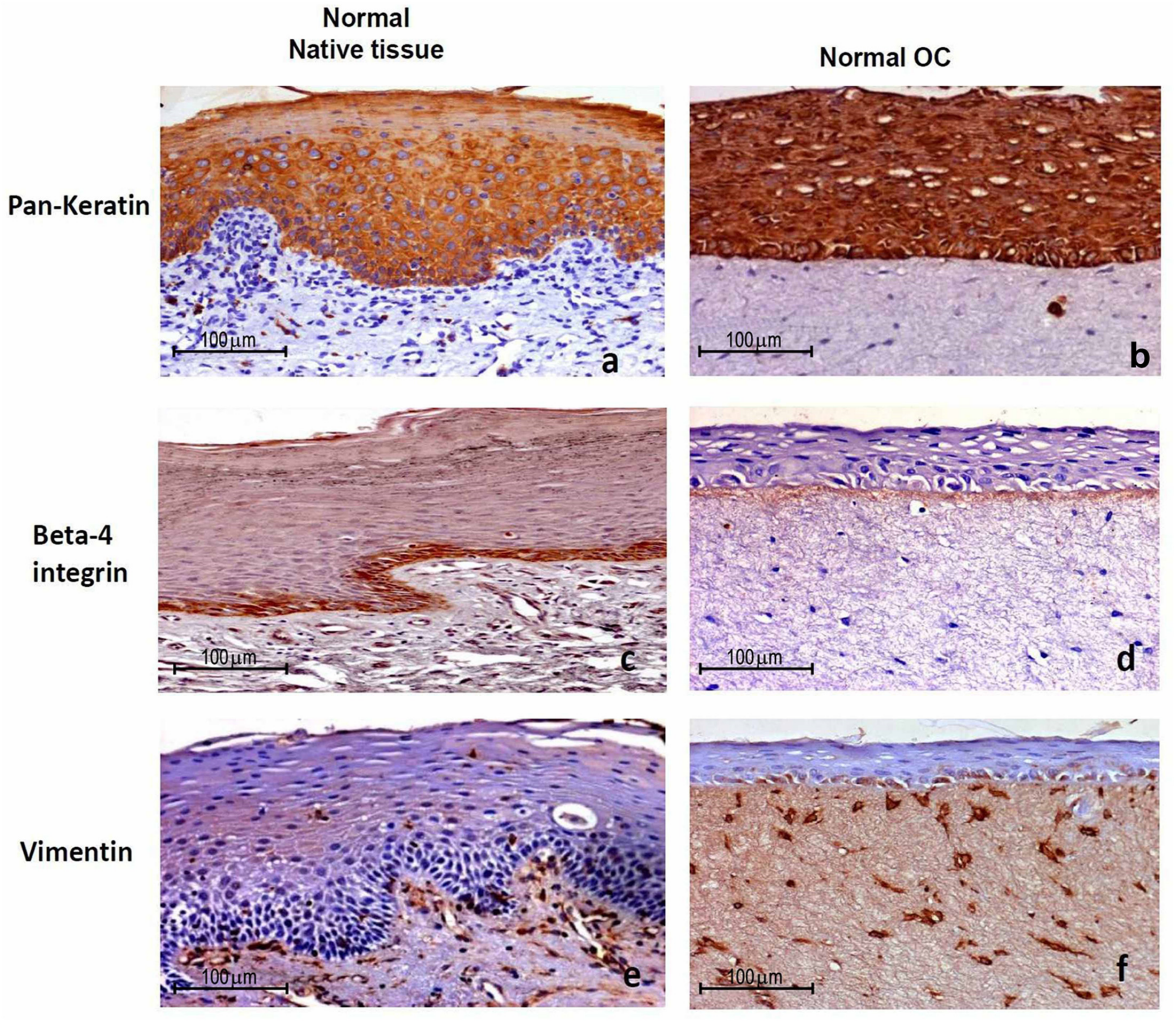
Validation of endogenous lineage phenotype. Immunohistochemistry of normal OC showing keratin (b) and vimentin (f) staining in the epithelium and stromal fibroblasts respectively and staining of integrin β4 (d) in the basement membrane as seen in native tissues (a, c, e). The experiments were performed in triplicates.

A schematic diagram of the protocol used to grow OCs at the air–liquid interface is shown in Fig 2A. These conditions favour the differentiation of keratinocytes and formation of a well-matured keratinized epithelium. Normal and dysplastic OCs grown either with or without fibroblasts displayed three major compartments of tongue tissue consisting of epithelium, underlying stroma, and the basement membrane in between connecting these two compartments (Fig 3). In contrast to normal and dysplastic Fib+ OC, malignant Fib+ OC did not show distinct compartments owing to their invasive phenotype (Fig 2Ba, 2Bc and 2Be). The morphology of all the three types of Fib+ OCs closely resembled the morphology of their native counterpart tissues. Although, normal Fib+ OCs showed multilayered epithelial growth, as compared to native epithelium (mean epithelial thickness of 142.8 ± 1.361 μm) their thickness was significantly less. A multilayered epithelium was also formed in Fib- OCs, this epithelium was significantly thinner than their Fib+ OC counterparts (Fig 2Bb, 2Bd and 2Bf). The normal Fib+ OCs showed a mean epithelial thickness of 69.51 ± 0.9661μm, while their counterparts Fib- OCs showed 30.19 ± 2.644 μm (*P* = 0.0002). Similarly, the dysplastic Fib+ OCs showed a mean epithelial thickness of 67.60 ± 4.513 μm, while Fib- OCs showed 43.25 ± 5.33 μm (*P* = 0.0252). The thickness of epithelium varied from patient to patient. Due to inadequate sample size, we cannot comment on the effect of clinicopathological parameters on growth and thickness of epithelium. Further, invasive depth analysis of malignant Fib+ OCs revealed that the depth of invasion of Fib+ OCs was 88.78 ± 4.767μm whereas in case of Fib- OCs, the cells remained in the epithelial compartment and hence there was no invasion (S1 Fig).

### Characterization of Endogenous Lineage Phenotype of *in-vitro* grown Tissues

Maintenance of the endogenous lineage phenotype (epithelial and mesenchymal) after *in-vitro* growth was checked by performing IHC staining. Immunostaining for pan-keratin was restricted to the epithelial compartment, vimentin staining was observed in the mesenchymal compartment and integrin β4 staining was located in the basement membrane of *in-vitro*-cultured normal tongue OC tissues. The staining pattern of keratin, vimentin and integrin β4 was comparable with their native-normal tongue tissue (Fig 3).

### Effect of Fibroblasts on Cell Proliferation and Differentiation

**Fig 4:**
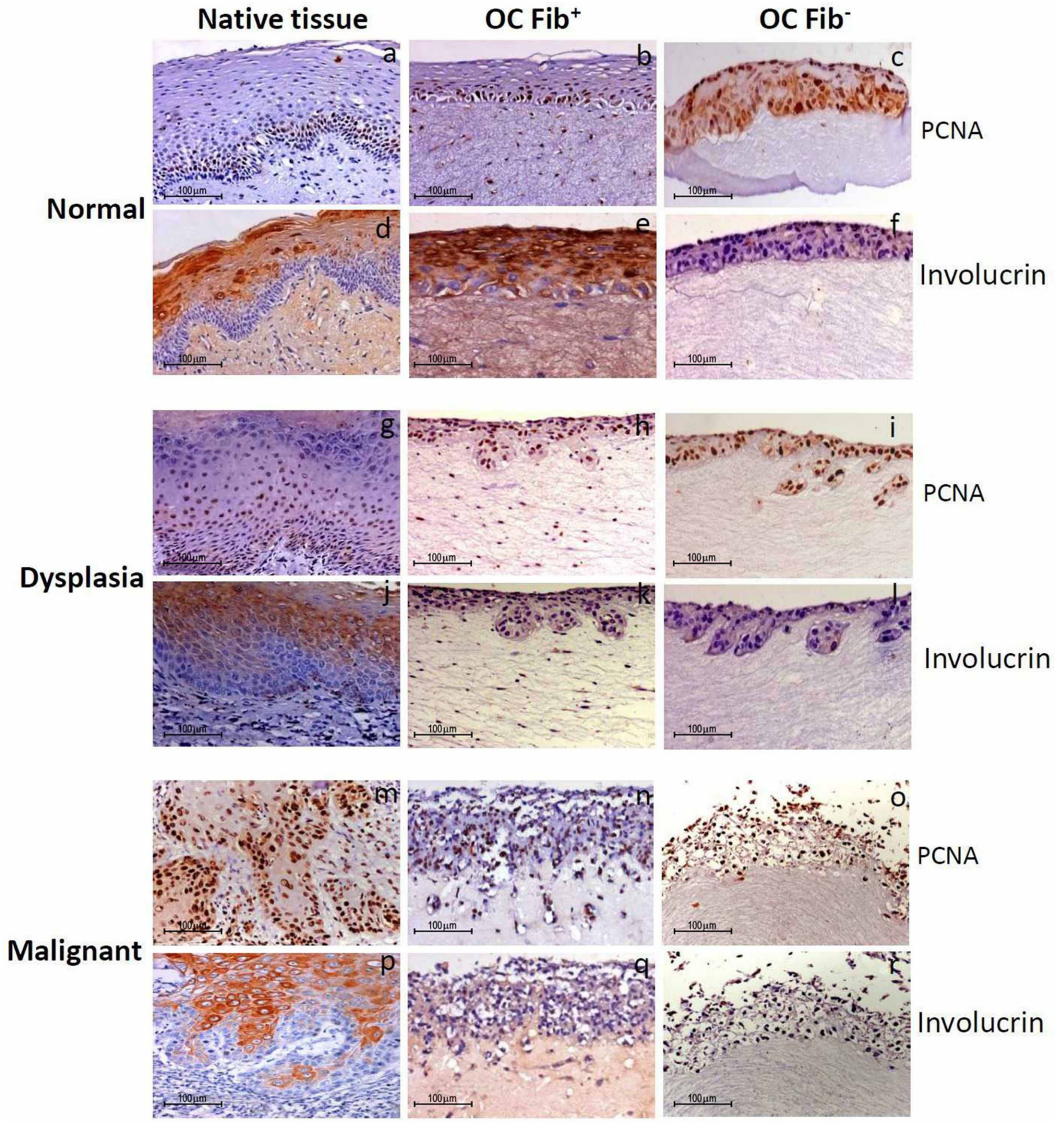
Cell proliferation and differentiation staining pattern in *in-vitro* grown tissues. Immunohistochemical staining for cell proliferation and differentiation specific proteins in Fib+ and Fib- OCs reconstructed from normal, dysplastic and malignant tongue tissues along with their respective native tissues: PCNA nuclear staining was seen in the basal proliferating cells of normal- native tissue, Fib+ and Fib- OCs (a, b, c) while cytoplasmic involucrin staining was seen in the upper differentiated cells of normal- native tissue, Fib+ and Fib- OCs (d, e, f). PCNA nuclear staining was seen in the basal as well as supra basal cells of dysplastic-native tissue, Fib+ and Fib- OCs (g, h, i) while cytoplasmic involucrin staining was seen in the native dysplastic tissue but not in Fib+ and Fib- OCs (j, k, l). PCNA staining was observed throughout the epithelium of malignant-native tissue, Fib+ and Fib- OCs (m, n, o), while involucrin staining was seen in the differentiated cells of malignant-native tissue but not in the Fib+ and Fib- OCs (p, q, r). The experiments were performed at least three times.

Next, we analyzed cell proliferation and differentiation characteristics of the three stepwise OC models grown with or without fibroblasts, and the staining pattern was compared with their respective native tissues. Proliferative cell nuclear antigen (PCNA), marker for cell proliferation, showed positive staining in the dividing cells for all normal, dysplastic, and malignant native tissues (Fig 4a, 4g and 4m), and its staining intensity, number of positive cells and their basal cell localisation was comparable with the Fib+ OCs -normal (Fig 4b), dysplasia (Fig 4h) and malignant (Fig 4n), but in Fib- OCs positive cells were localised throughout the epithelium (Fig 4c, 4i and 4o). On the other hand, involucrin, a marker of epithelial differentiation showed comparable staining pattern in normal Fib+ OCs and their respective native tissues (Fig 4d and 4e). However, OCs developed from dysplastic and malignant tissues, irrespective to presence or absence of fibroblasts, showed sparse to negative staining (Fig 4f, 4k, 4l, 4q and 4r). In contrast, their respective native tissues showed positive staining in the terminally differentiated cells (Fig 4j and 4p).

### Role of Fibroblasts in Expression and Localization of Desmosomal Anchoring Proteins

Since the desmosomal proteins play a major role in the process of tumour cell invasion, we analyzed the expression of major desmosomal proteins-desmoplakin, desmoglein and plakoglobin, in all the three OC models. Furthermore, we compared their staining intensity between Fib+ OCs and Fib- OCs using semi-quantitative analysis. All the proteins showed localization on the cell membrane and in the cytoplasm of epithelial cells.

#### Desmoplakin

**Fig 5:**
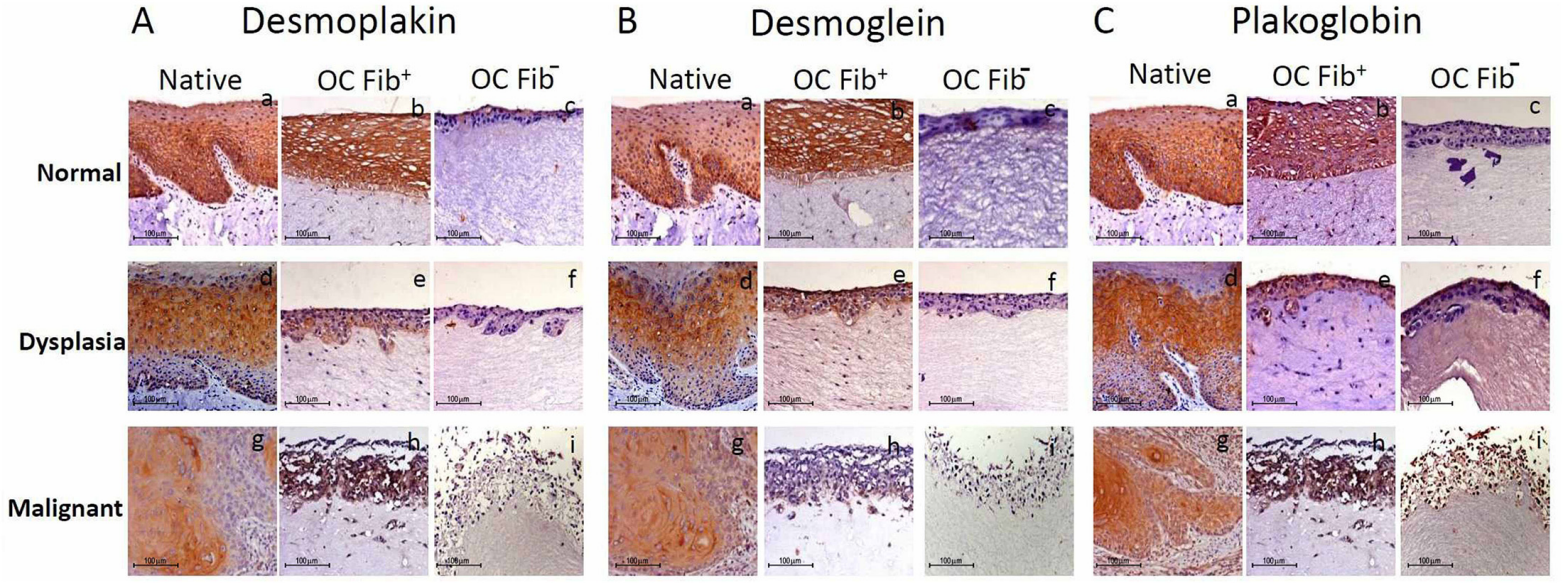
Immunohistochemical staining pattern of desmosomal proteins in *in-vitro* grown tissues. IHC staining of desmoplakin (Aa-i), desmoglein (Ba-i) and plakoglobin (Ca-i) in Fib+ and Fib- OCs reconstructed from normal, dysplastic and malignant tongue tissues along with their respective native tissues. All three types of native tissues- normal, dysplastic and malignant showed similar immunolocalization and staining intensity for desmoplakin (Aa, b, d, e, g, h), desmoglein (Ba, b, d, e, g, h) and plakoglobin (Ca, b, d, e, g, h) in comparison with Fib+ OCs. While, Fib- OCs showed weak staining throughout all epithelial layers for desmoplakin (Ac, f, i), desmoglein (Bc, f, i) and plakoglobin (Cc, f, i). The experiments were performed at least three times.

In native tissues of normal, the staining was intense in basal and para-basal cells, and upper differentiated cells were moderately stained (Fig 5Aa), while in dysplastic tissue, basal cells were moderately stained, and upper differentiated cells were strongly stained (Fig 5Ad). In native malignant tissue, the weak staining was seen throughout the section (Fig 5Ag). Normal Fib+ OC showed intense staining throughout the section (Fig 5Ab), while dysplastic and malignant OCs showed moderate to weak staining throughout the sections (Fig 5Ae and 5Ah). Fib- OC showed weak staining in normal, dysplastic, and malignant OCs (Fig 5Ac, 5Af and 5Ai).

#### Desmoglein

The staining pattern was similar to that of desmoplakin, more intense in the basal compartment of normal native tissue, while in the dysplastic tissue, it was localized in the upper spinous cells, and in malignant tissue, it was similar throughout the epithelium (Fig 5Ba, 5Bd and 5Bg). In normal and dysplastic Fib+ OCs, the staining intensity was strong and uniform throughout the epithelium (Fig 5Bb and 5Be), whereas in malignant Fib+ OCs and all Fib- OCs, the staining intensity was weak (Fig 5Bh, 5Bc, 5Bf and 5Bi).

#### Plakoglobin

In normal native tissues, the basal cells were intensely stained, while in dysplastic tissues, the suprabasal cells were most intensely stained, and in the malignant tissue, there was an accumulation of staining in the tumour islands (Fig 5Ca, 5Cd and 5Cg). In contrast to this, all three types of Fib+ OCs showed the same intensity throughout the epithelium (Fig 5Cb, 5Ce and 5Ch), while Fib- OCs showed a very weak staining intensity (Fig 5Cc, 5Cf and 5Ci).

**Fig 6:**
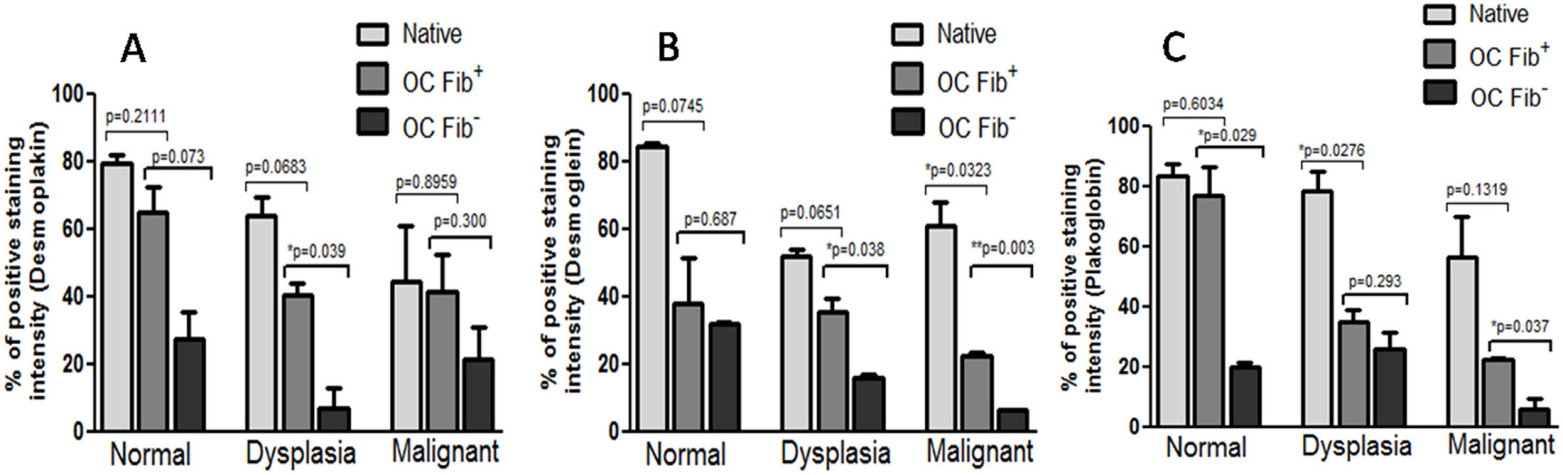
Analysis of immunohistochemistry staining intensity using ImageJ. Graphical representation of percentage of positive staining intensity of desmoplakin (A), desmoglein (B) and plakoglobin (C). IHC staining intensity score was calculated using IHC profiler plugin in ImageJ software. Statistical analysis was done between cultures grown with and without fibroblasts using two-tailed unpaired *t-test* and represented as p values. The experiments were performed in triplicates and data are presented here as mean ± standard errors.

Furthermore, semi-quantitative analysis revealed that the staining intensity of desmosomal proteins, viz., desmoplakin (Fig 6A), desmoglein (Fig 6B), and plakoglobin (Fig 6C) was higher in normal, dysplastic and malignant Fib+ OCs when compared with their respective Fib- OCs. However, only dysplastic Fib+ OCs and Fib- OCs showed significant decrease in desmoplakin staining intensity (*P* = 0.0397), while in case of desmoglein staining intensity, both dysplastic (*P* = 0.038) and malignant (*P* = 0.003) OCs showed significant decrease. Plakoglobin staining intensity was significantly reduced in normal (*P* = 0.029) and malignant (*P* = 0.037) Fib+ OCs when compared with Fib- OCs.

### Role of Fibroblasts in Maintenance of Ultrastructure of Desmosomes

**Fig 7:**
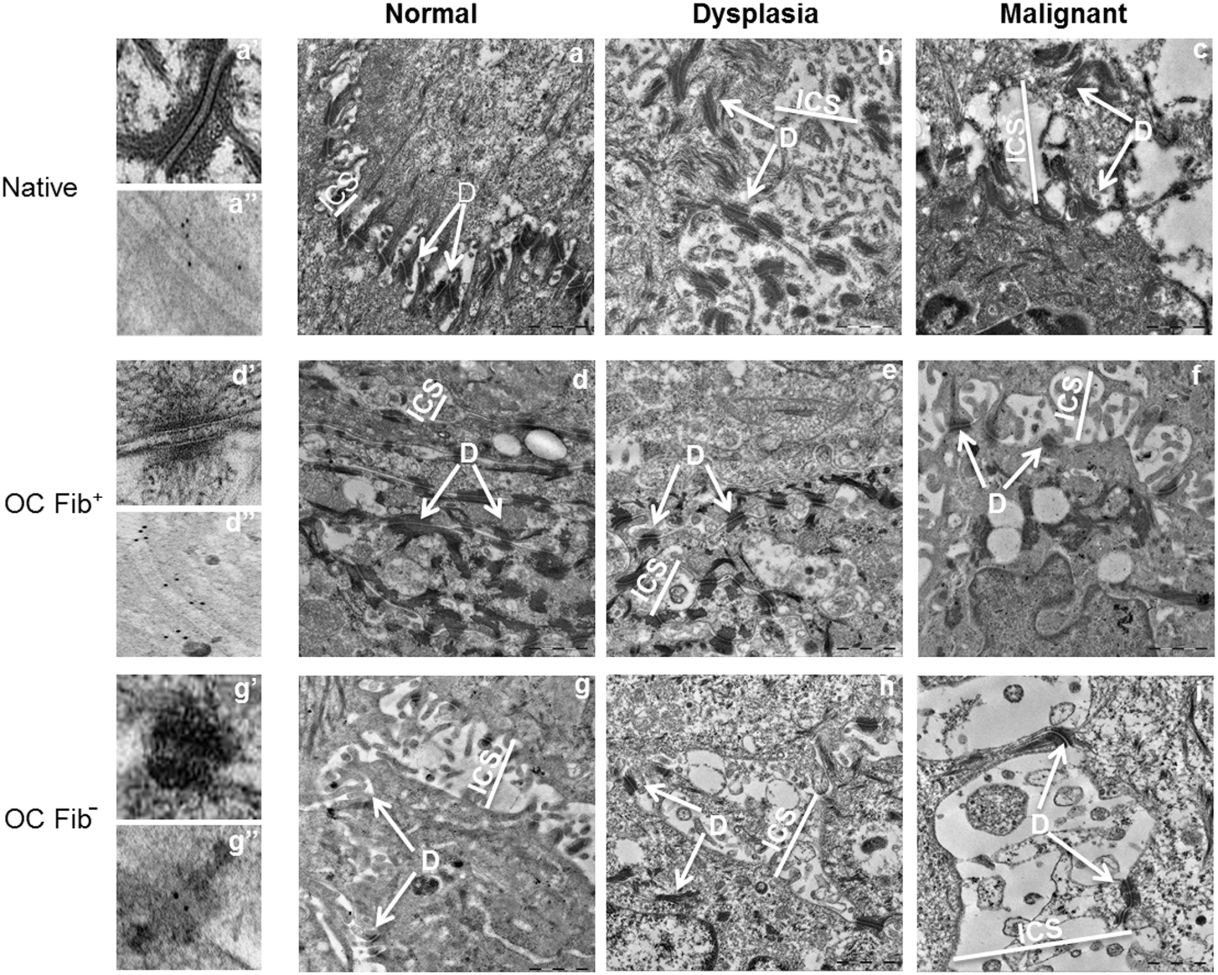
Ultrastructural analysis of altered desmosomal assembly. Electron micrographs depicting desmosomes (D) and intercellular spaces (ICS) in native tissues (a-c) along with Fib+ OCs (d-f) and Fib- OCs(g-i). Native tissue and Fib+ OCs show similar ultrastructure having widened intercellular spaces (ICS) and reduced number of desmosomes as disease stage progressed, whereas Fib- OCs showed complete collapse of ultrastructure with more widened ICS and reduced desmosomes even at normal stage. Normal desmosome structure was seen in normal-native tissues as well as Fib+ OCs (a’, d’) but in normal-Fib- OCs desmosomes were found to be in aggregated forms (g’). Desmosome structures were confirmed by immunogold labeling in normal native tissue (a”) normal Fib+ OC (d”) and normal Fib- OC (g”). Bars 1 μm.

In normal Fib+ OCs, two-electron dense plaques on the adjacent cell membranes could be observed by TEM. These plaques interacted with the intermediate filaments toward cytoplasmic side mimicking the desmosome structures observed in the native tissues (Fig 7a’ and 7d’). Immunogold labeling was detected on the desmosome structures of both normal native tissue and normal Fib+ OCs as shown in Fig 7a” and 7d” respectively. The ultrastructure of desmosomes in Fib- OCs was largely conspicuous. Even though immunogold labeling confirmed these structures as desmosomes (Fig 7g”), their morphology was different from the desmosomes formed in native tissue. Instead, the structures showed electron dense clusters without formation of two plaques along with the core (Fig 7g’).

**Fig 8:**
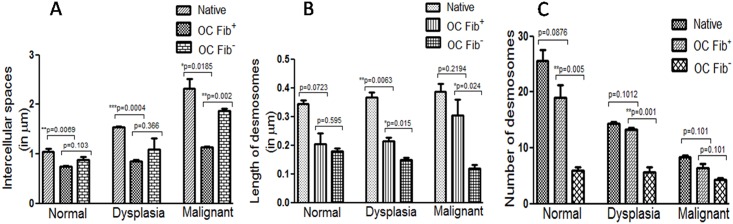
Quantitative analysis of alterations in desmosomal assembly using iTEM software. Graphical representation showing variation in desmosome number (A), their length (B) and intercellular spaces (C) in all three OC models along with their respective native tissues. Statistical anaylsis was drawn in between Fib+ and Fib- OCs and represented as p values. The experiments were performed at least three times and data represented here as mean ± standard errors.

Next, ultrastructural changes in number and length of desmosomes, and intercellular spaces were compared between Fib+ and Fib- OCs. In normal native tissues, desmosomes were located along the side of two adjacent cell membranes with minimal intercellular spaces (Fig 7a). In dysplastic native tissues, they were dislocated from cell membrane with wider intercellular spaces, and in malignant tissues, the desmosomes were found hanging on one side of cell membrane with even more widened intercellular spaces (Fig 7b and 7c). Similar observations were found in dysplastic and malignant Fib+ OCs (Fig 7d–7f). However, there was major difference in the localization of desmosomes and intercellular spaces of dysplastic and malignant Fib- OCs (Fig 7g–7i). We found significant difference in the desmosome number (Fig 8A), their length (Fig 8B), and intercellular spaces (Fig 8C) between Fib+ and Fib- OCs.

## Discussion

There is a need of some reliable *in-vitro* model system that can reproducibly reflect the multiple stages of oral tumorigenesis for the better understanding of the process of neoplastic progression. Previous studies have shown *in-vitro* 3D reconstruction of oral mucosal tissues with features closely mimicking the native counterpart [8, 10, 34]. Researchers have also developed *in-vitro* 3D models and studied invasive characteristics of normal versus malignant keratinocytes and fibroblasts [8, 35]. However, to our knowledge we have established for the first time, *in-vitro* 3D models representing different progressive steps of tongue tumorigenesis. Further, we have focused on the role of fibroblasts in the maintenance of cell desmosomal anchoring assembly at protein as well as ultrastructural level.

Although we performed direct explant culture technique and dispase enzyme digestion technique for the isolation of keratinocytes and fibroblasts from tongue tissues, we obtained better yield of both types of cells by former technique. Low success rate (<20%) of *in-vitro* 3D model was primarily because of bacterial contamination, which could be attributed to the patients’ poor oral hygiene. Another reason was that all the three types of OCs- normal, dysplastic and malignant have been grown in a true pair having fibroblasts and keratinocytes/carcinoma cells from the same patient. This was done to avoid the influence of cells from different environment. Other groups have used keratinocytes and fibroblasts in true pairs [8, 35] as well as originating from different milieu for the growth of *in vitro* 3D models [21, 23, 36].

Histology of *in-vitro* grown cultures revealed three distinct compartments, similar to native tissue that is epithelium, underlying connective tissue and in between basement membrane. Further, we confirmed that our co-culture model system retained the original epithelial/mesenchymal endogenous phenotype indicating that the exogenous culture conditions did not affect the basic lineage (epithelial/mesenchymal) characteristics of the cultured cells used for *in-vitro* reconstitution of tissues. We found variable thickness of OCs generated from tissues obtained from different patients. Technical repeats of OCs using same keratinocytes and fibroblasts did not yield any difference in the epithelial thickness.

There are contrasting reports on the influence of connective tissue on gene regulation and functional modifications of epithelium. According to some researchers, epithelial differentiation is an inherent feature of these cells, which is not dependent on the underlying mesenchymal tissue [37, 38], whereas others have observed an effect of mesenchymal tissue on epithelial cell differentiation [39, 40]. It is known that fibroblasts secrete several growth factors and interleukins, such as interleukins 1, 6, and 8, transforming growth factor -a and b, granulocyte macrophage colony stimulating factor, as well as members of the fibroblasts growth factor family [8]. These diffusible factors may modulate epithelial growth and differentiation. As reported previously in native oral mucosa, these growth factors could also be provided by other neighboring cells, such as gamma delta T lymphocytes [41, 42]. It has also been documented that, serum-free medium supplemented with growth factors as well as the number and the functional state of fibroblasts affect the epidermal differentiation program [43]. Morphometry analysis showed difference between the epithelial thickness of native tissue and Fib+ OCs which could be attributed to the fact that for the growth of OC cultures we have used only collagen and fibroblasts as stromal components. However, stroma is composed not only of fibroblasts and collagen but also of fibrous connective tissue, capillaries, macrophages, and extra cellular matrix [44, 45]. Hence, additional exogenous stromal components might be required for the optimum stratification of *in-vitro* grown tongue tissues.

Dysplastic Fib+ OCs showed moderate loss of polarity of basal cells whereas malignant Fib+ OCs showed severe loss, resulting in an infiltration of scattered tumour cells towards the stroma and enhanced depth of invasion, faithfully simulating the phenotype of their respective native tissues. However, the malignant Fib- OCs remained in the epithelial compartment, pinpointing that the established Fib+ OCs models showed resemblance with their native tissue but not Fib- OCs. This corroborates with previous studies on oral carcinogenesis [5, 8] and demonstrates that fibroblasts are essential for tumour cell invasion. Our results also support the hypothesis that fibroblasts may have an essential role not only in the late processes of tumour invasion but also in the early processes of cell transformation [19]. Nonetheless, in addition to fibroblasts, various collagens, laminins, cytokines and non-vital stromal and inflammatory cells are responsible for invasion as shown using myoma model [32, 46].

Expression of the cell differentiation-specific marker, involucrin, was found in normal Fib+ OCs but not in normal Fib- OCs, indicating a role of fibroblasts in differentiation of normal tongue epithelium in OC cultures. But in case of dysplastic and malignant tissues irrespective to presence or absence of fibroblasts, immunoreactivity of involucrin was very weak. This observation could be attributed to the fact that, although in *in-vitro* normal Fib+ OCs, the fibroblasts embedded in collagen matrix contributed to the regulation of cell differentiation function, but as the disease stage increased, their control on cell differentiation may have decreased, as previously suggested for buccal carcinogenesis [24].

Although, there was no difference in cell proliferation either in presence or absence of fibroblasts across the three OCs-normal, dysplastic and malignant, we did find difference in their localization. As seen in normal native and normal Fib+ OCs, PCNA positive cells were restricted mostly to the basal cell layer, while in Fib- OCs the positive cells were distributed randomly throughout all epithelial layers. It appears that even though, there is no difference in the proliferation index, there is a difference in the epithelial thickness of Fib+ versus Fib- OCs. Of notice this observation could be attributed to the fact that in Fib- OCs, cell proliferation is the only major factor contributing to epithelial thickness, whereas in Fib+ OCs, apart from cell proliferation, fibroblasts secreted factors contributed in cell differentiation which in turn modulated the thickness of the epithelium [4, 8, 47]. Previous reports have shown that fibroblasts circumvents the cell death program of reconstituted epithelium by decreasing cell death in the basal cells and by promoting terminal differentiation in suprabasal cells, a pattern normally found in native oral mucosa, thereby increasing epithelial thickness by modulating proliferation [8].

Although the role of stromal microenvironment, especially stromal fibroblasts, has been shown in the growth and differentiation of epithelium, their role in maintaining the molecular and structural integrity of desmosomal junctions has not yet been elucidated. TEM observations revealed that desmosome number and their length was reduced in Fib- OCs when compared with Fib+ OCs, and the intercellular spaces were wider in Fib- OCs when compared with Fib+ OCs. The desmosome structure appeared as previously described in the native tongue epithelium and Fib+ OCs with two dense plaques on neighbouring membranes. However, in some of the epithelial cells of Fib- OCs, desmosomes showed dense aggregates in place of plaques. We presume that those could be aggregated form of plaque proteins. These observations suggest that the stromal fibroblasts play a role in maintaining molecular and ultrastructural integrity of desmosomal junctions, even though there is no direct contact between them. This effect might be exerted by importing fibroblasts secreted growth factors and cytokines by upper epithelium as suggested previously [8, 42]. This also explains that the expression and localization of desmosomal proteins as well as the ultrastructure of desmosomal assembly was found deregulated in Fib- OCs as compared to Fib+ OCs.

It is important to note that these 3D OC models have number of shortcomings such as: (1) the amount of tissue obtained from one donor is limited, (2) potential contamination with pathogens/bacteria owing to poor oral hygiene, (3) donor to donor variability, and (4) the success rate is poor. We anticipate that, in spite of these shortcomings, over a period of time, these models will bridge the gap between monolayer cell cultures and animal models. Presently, our laboratory is investigating the utility of these models to identify alterations occurring in basal lamina and hemidesmosomes during the process of oral tumorigenesis.

To conclude, reproducible *in-vitro* tissue models representing major steps of human tongue tumorigenesis have been established. Our findings indicate that, stromal fibroblasts have an influence on the maintenance of molecular and structural integrity of desmosomal anchoring junctions. *In-vitro* reconstituted tongue tissue models will be useful tool in addressing a broad range of unanswered questions in oral pathogenesis and, especially, in the multistep process of tongue tumorigenesis. In future, this model might be a useful biological standardized system for testing anticancer drugs for personalised treatment.

## Supporting Information

S1 Fig Analysis of invasive depth of malingnant OCs. Pan cyto-keratin immunostained images of day 6 and 16 malignant Fib- OCs (Aa, b) and Fib+ OCs (Ac, d). The dashed line (yellow) represents lower surface of non-invasive cell layer. The distance of the deepest invading cell from the lower surface of this non-invasive cell layer was measured in immunostained Fib+ OC at day 16 using ImageJ software (B). The results consist of three measurements each of thirteen samples. There was no invasion in Fib- OCs. Bars 50 μm.
(TIF)

S1 Table Clinicopathological information of patients with dysplasia and OSCC. Table describing age, gender, tobacco habits and clinicopathological information of patients with dysplasia (A) and OSCC (B)
(DOCX)
