# Supplementary figures and images for: Establishment of 3D Co-Culture Models from Different Stages of Human Tongue Tumorigenesis: Utility in Understanding Neoplastic Progression
Source: PLoS One. 2016 Aug 8;11(8):e0160615. doi: 10.1371/journal.pone.0160615 (PMC4976883; doi:10.1371/journal.pone.0160615)

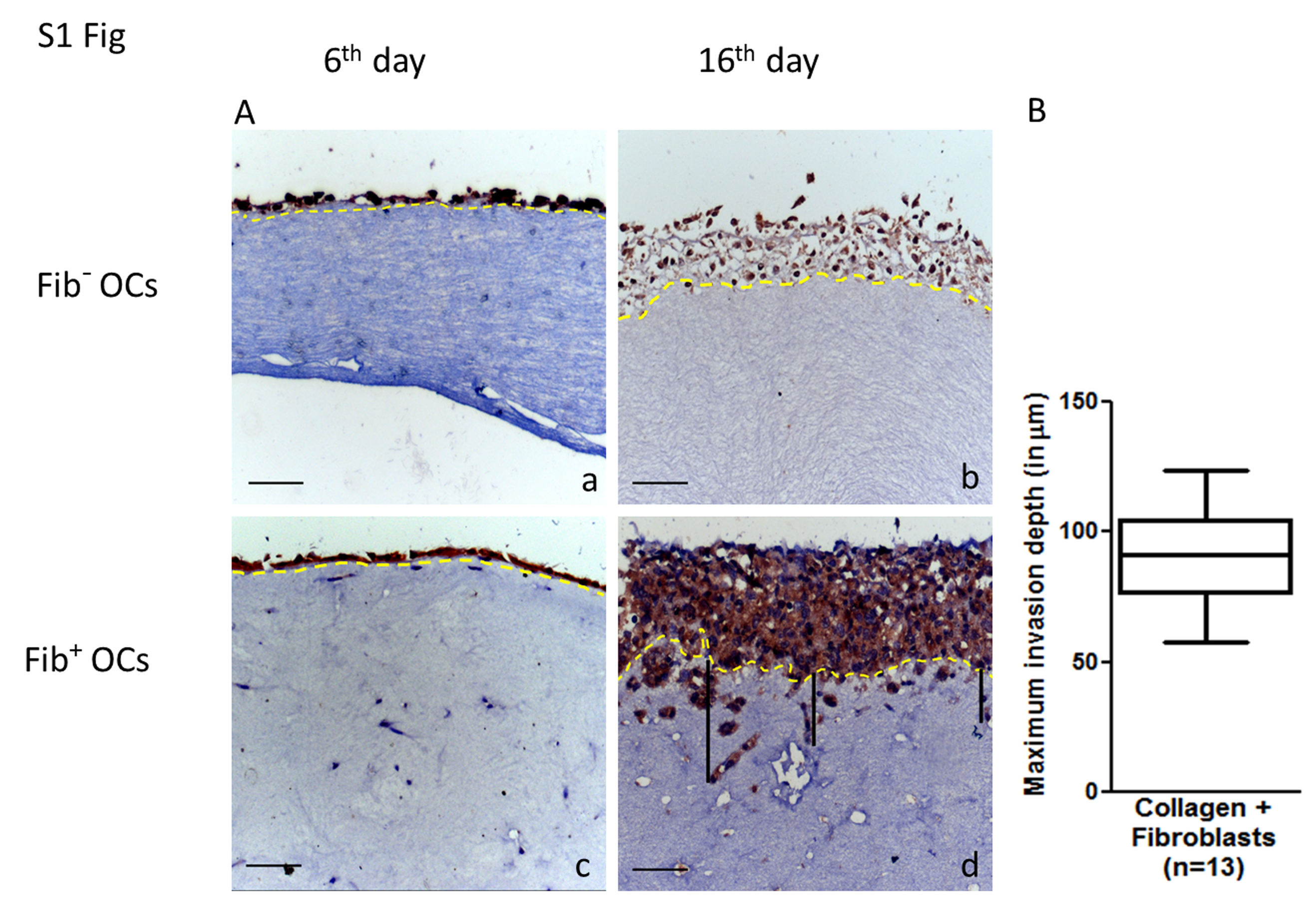

Supplement: S1 Fig — Pan cyto-keratin immunostained images of day 6 and 16 malignant Fib- OCs (Aa, b) and Fib+ OCs (Ac, d). The dashed line (yellow) represents lower surface of non-invasive cell layer. The distance of the deepest invading cell from the lower surface of this non-invasive cell layer was measured in immunostained Fib+ OC at day 16 using ImageJ software (B). The results consist of three measurements each of thirteen samples. There was no invasion in Fib- OCs. Bars 50 μm. (TIF) [file pone.0160615.s001.tif]
