# Supplementary material for: Establishment of 3D Co-Culture Models from Different Stages of Human Tongue Tumorigenesis: Utility in Understanding Neoplastic Progression
Source: PLoS One. 2016 Aug 8;11(8):e0160615. doi: 10.1371/journal.pone.0160615 (PMC4976883; doi:10.1371/journal.pone.0160615)
Supplement: S1 Table — Table describing age, gender, tobacco habits and clinicopathological information of patients with dysplasia (A) and OSCC (B) (DOCX) [file pone.0160615.s002.docx]

S1 Table.Table describing age, gender, tobacco habits and clinicopathological information of patients with dysplasia and OSCC.

| Dysplasia | Age (years) | Gender | Tobacco Habits | Dysplastic Grade |
| --- | --- | --- | --- | --- |
| 1 | 49 | M | S +C | Mild |
| 2 | 55 | M | S +C | Moderate |
| 3 | 58 | F | S | Mild |
| 4 | 60 | M | C | Moderate |
| 5 | 47 | M | C | Moderate |

A:Dysplasia

B: OSCC

| Tumour/normal | Age (years) | Gender | TobaccoHabits | Tum-our stage | Nodal Metastasis | Bone Involvement | Perineural Invasion | Lympho vascular invasion | Perinodal extension | Skin involve-ment | Differen-tiation |
| --- | --- | --- | --- | --- | --- | --- | --- | --- | --- | --- | --- |
| 1 | 46 | M | S +C | T3 | N2 | No | No | No | Yes | No | PD |
| 2 | 36 | M | S +C | T2 | N1 | No | No | No | Yes | No | MD |
| 3 | 56 | M | S +C | T4 | N1 | No | No | No | Yes | No | PD |
| 4 | 57 | M | S | T2 | N2 | No | Yes | No | Yes | No | PD |
| 5 | 48 | M | S +C | T3 | N1 | No | No | No | Yes | No | PD |
| 6 | 30 | M | S +C | T4 | N2 | No | No | No | Yes | No | WD |
| 7 | 53 | M | C | T4 | N2 | No | Yes | No | Yes | No | MD |
| 8 | 52 | M | C | T2 | N0 | No | No | No | No | No | MD |
| 9 | 35 | M | S +C | T3 | N1 | No | No | No | Yes | No | MD |
| 10 | 47 | M | S | T4 | N2 | No | Yes | No | Yes | No | MD |
| 11 | 40 | M | C | T2 | N0 | No | No | No | No | No | MD |
| 12 | 32 | M | S +C | T4 | N1 | No | Yes | No | Yes | No | PD |
| 13 | 49 | M | S +C | T2 | N2 | No | Yes | No | Yes | No | PD |

M: Male, F: Female, S: Smoking, C: Chewing, N0: No lymph-node spread, N1 and N2: lymph-node spread, WD: Well differentiated, MD: Moderately differentiated, PD: Poorly differentiated.
